# To zoom or not to zoom – the training of communicative competencies in times of Covid 19 at Witten/Herdecke University illustrated by the example of “sharing information”

**DOI:** 10.3205/zma001376

**Published:** 2020-12-03

**Authors:** Katharina Knie, Laura Schwarz, Clarissa Frehle, Heike Schulte, Angelika Taetz-Harrer, Claudia Kiessling

**Affiliations:** 1 Universität Witten/Herdecke, Fakultät für Gesundheit, Lehrstuhl für die Ausbildung personaler und interpersonaler Kompetenzen im Gesundheitswesen, Witten, Germany; 2 Universität Witten/Herdecke, Fakultät für Gesundheit, Studiendekanat Humanmedizin, Witten, Germany; 3 Universität Witten/Herdecke, Fakultät für Gesundheit, Lehrstuhl für Didaktik und Bildungsforschung im Gesundheitswesen, Witten, Germany

**Keywords:** medical education, communication skills training, sharing information, digital teaching, inverted classroom, covid 19 pandemic

## Abstract

Since October 2018, a longitudinal communication curriculum for medical students has been implemented at Witten/Herdecke University. In the summer semester 2020, the concept for the 4^th^ preclinical semester included a practical training on “sharing information”, which consisted of three two-hour face-to-face sessions with simulated patients (SP). Due to the Covid 19 pandemic, teaching was changed to an inverted classroom concept combining asynchronous and synchronous teaching. The students worked at the beginning of the semester on an e-learning module of the learning platform docCom.deutsch on the topic “sharing information” using reflection and processing tasks. In two digital sessions, the students then were able to practice discharge interviews and discussions about risk communication illustrated by the example of screening methods for cancer prevention. In the first zoom session, students practiced in role-plays among themselves. In the second zoom session, they practiced with SP. The evaluation results revealed that 76% of the responding students considered working with the e-learning module as a good preparation for the interviews. According to the evaluation results, satisfaction with the Zoom meeting including SP contact was slightly higher than those with role-plays among themselves. Although the group atmosphere was rated by all responding students as conducive to learning, almost half of them confirmed that using Zoom significantly impaired the atmosphere (47%). In retrospect, the conversion of the communication training to a digital format worked better than expected from both the perspective of teachers and students. The students explicitly appreciated working with SP. From the teachers’ perspective, some specific aspects of successful communication were difficult to reflect on, e.g. non-verbal communication. The use of e-learning as a preparation for practical exercises has proven successful and will be continued in the future.

## Zusammenfassung

Seit Oktober 2018 wird an der Universität Witten/Herdecke ein longitudinales Kommunikations-Curriculum für Medizinstudierende implementiert. Im Sommersemester 2020 sah das Konzept für das 4. Semester ein Training zum Thema „Informationen weitergeben“ vor, das aus drei zweistündigen Präsenzveranstaltungen mit Simulationspatient*innen (SP) bestand. Aufgrund der Covid 19-Pandemie wurde der Unterricht auf ein inverted classroom Konzept mit asynchroner und synchroner Lehre umgestellt. Die Studierenden bearbeiteten zuerst anhand von Reflexions- und Bearbeitungsaufgaben ein e-learning Modul der Lernplattform docCom.deutsch zum Thema „Informationen weitergeben“. In zwei digitalen Veranstaltungen hatten die Studierenden dann die Gelegenheit, Entlassungsgespräche und Gespräche zur Einschätzung von Screeningverfahren zur Krebsvorsorge zu üben, im 1. Zoom-Termin in Rollenspielen untereinander, im 2. Zoom-Termin mit SP. In der Evaluation wurde das Arbeiten mit dem e-learning Modul von 76% der Studierenden, die an der Evaluation teilnahmen, als gute Vorbereitung auf die Gespräche eingeschätzt. Bei den Zoom-Terminen war die Zufriedenheit mit den Gesprächen mit SP etwas größer als mit den Rollenspielen untereinander. Obwohl die Gruppenatmosphäre von allen antwortenden Studierenden als lernförderlich eingeschätzt wurde, bestätigte fast die Hälfte, dass die Nutzung von Zoom die Atmosphäre deutlich beeinträchtigt habe (47%). Die Umstellung des Kommunikationstrainings auf ein digitales Format hat rückblickend aus Sicht der Dozierenden und Studierenden besser funktioniert als erwartet. Explizit gelobt wurde von den Studierenden der Einsatz der SP. Aus Sicht der Dozierenden waren einzelne Aspekte einer gelungenen Kommunikation schwierig zu reflektieren, z.B. die nonverbale Kommunikation. Der Einsatz von e-learning als Vorbereitung auf das praktische Üben hat sich bewährt und wird auch zukünftig beibehalten.

## 1. Background

Since the winter semester 2018/19, a longitudinal communication curriculum has been implemented at the University of Witten/Herdecke (UW/H) as part of the model study programme Medicine 2018+ [1], [2]. The curriculum is designed to cumulatively develop communication competence, starting in the first two preclinical semesters with the training of basic competencies (e.g. building relationships, dealing with emotions, providing structure). In the third pre-clinical semester, communication skills are trained in the context of history taking with simulated patients (SP) and assessed in an OSCE station. The concept for the 4th pre-clinical semester, which was planned to be implemented for the first time in the summer semester 2020, focusses on the training of “sharing information”. The initial plan included three two-hour sessions at the beginning, middle and end of the semester with SP. 

Due to the Covid 19 pandemic, it was decided at the UW/H in March 2020 to switch teaching to digital formats. The faculty recommended Zoom for the technical implementation, since Zoom was free of charge for educational institutions and could be used relatively trouble-free at the beginning of the semester. Therefore, an inverted classroom concept [3] with a combination of asynchronous and synchronous teaching was developed for the communication curriculum. 

## 2. Project description

The asynchronous teaching included working on an e-learning module in preparation for the synchronous teaching, offering the possibility of time flexibility. The synchronous teaching included practical training in the digital space. In terms of learning theory, this could be justified by Hargie's [4] considerations that clinical communication is a social competence and that learners benefit from building a foundation of procedural knowledge before applying it in exercises. 

At the beginning of the semester, students received compulsory written reflection and processing tasks by e-mail for the e-learning module 10 “Sharing Information” [5] of the learning platform docCom.deutsch [https://doccom.iml.unibe.ch/startseite/] for asynchronous self-study. The reflection task served to activate previous experiences with patient contacts. The students then completed the e-learning module in writing with a structured task assignment. The tasks served to deal in depth with the contents of the e-learning module (e.g. with the techniques “ask-tell-ask” or “book metaphor”, supporting and hindering factors in the process of information transfer, wishes and needs of patients for information transfer). The student’s elaborations had to be submitted by email before the first Zoom session. The concept of synchronous teaching in Zoom meetings was adapted to the learning needs based on the remaining open questions from the sighted student texts. In addition, the students were sent two more voluntary exercises by email: the reformulation of several closed questions into open questions and the translation of a discharge letter into plain language [idea according to [6]. 

The obligatory Zoom sessions were carried out in seven small groups of 6-7 students each with one facilitator. During the first Zoom session, the students created a checklist for the preparation of a discharge interview. Afterwards, they practiced a discharge interview in role-plays among themselves, based on the discharge letter they had received in advance for practice purposes. In the role-plays, the students adopted one of three different perspectives: doctor, patient, and observer. After the role-plays, they provided feedback from that specific perspective.

The second Zoom session addressed the topic of risk communication [7], [8]. The students were again able to practice the sharing of information using the examples of screening procedures for breast cancer and prostate cancer prevention. Prior to this, they received information material for voluntary self-study and a lecture on the methodological, statistical and ethical principles of cancer screening procedures.

After the discussion of open questions and the joint theoretical preparation, two students conducted a doctor-patient interview for risk communication with an SP. The other students observed the conversations and provided feedback subsequently. The session ended with collecting feedback on the course concept. 

## 3. Results

The evaluation was carried out using an online questionnaire and included 16 closed questions (7-step scale from 0=“don't agree at all” to 6=“fully agree”) and two open questions. The response rate was 21 out of 42 students, with 17 answers being evaluable (40%). Table 1 (Tab. 1) summarizes the most important evaluation results.

## 4. Discussion

The challenge in designing the courses of the past semester was to quickly convert an analogue communication skills training to an exclusively digital concept. Teaching involved the use of asynchronous and synchronous elements that built on each other and were oriented to the students’ learning needs. Specific communication skills, which are helpful for sharing information with patients, were practiced in role-plays with and without SP illustrated by the examples of discharge interviews and risk communication. The evaluation results revealed that the students perceived the teaching units very positively and confirmed that they were able to improve their communication skills. Most of the responding students evaluated working with the e-learning module also very positively. In the Zoom sessions, the assumed benefit of the discussions with SP was greater than for the Zoom session without SP. Although the group atmosphere in the Zoom meetings was considered by all responding students to be conducive to learning, almost half of them confirmed that using Zoom had significantly impaired the atmosphere. From the perspective of the teachers, the discussion of some specific communication skills was difficult, e.g. body language and eye contact. However, teaching in digital space was more effective than expected. Students appreciated explicitly the participation of the SP at the second Zoom event in the open evaluation comments.

A disadvantage of the new format was that not every student was able to talk to a SP. An advantage was the thorough theoretical preparation by means of e-learning, which would not have taken place in the original analogous concept. The change of the concept was an enormous additional effort for the teachers (adaptation of didactic concepts, development of new worksheets, review of the processed worksheets, and familiarization with Zoom). It remains an open question, how this can be reflected in the calculation of the regular teaching obligations. 

## 5. Conclusions

The new experiences led to a rethinking of old concepts and to an expansion of our didactic repertoire. Digital teaching with regard to communication skills training has its place – if sensibly integrated – especially the asynchronous preparation by means of e-learning. Zoom-meetings offer the possibility to conduct teaching independently of location. Especially in the decentralized clinical phase of studies, this advantage can be used to enable teaching that would otherwise require travel time for students or teachers. The establishment of a supporting group atmosphere as well as the reflection of non-verbal aspects of conversation in digital space, however, requires further consideration.

## Competing interests

The authors declare that they have no competing interests. 

## Figures and Tables

**Table 1:**
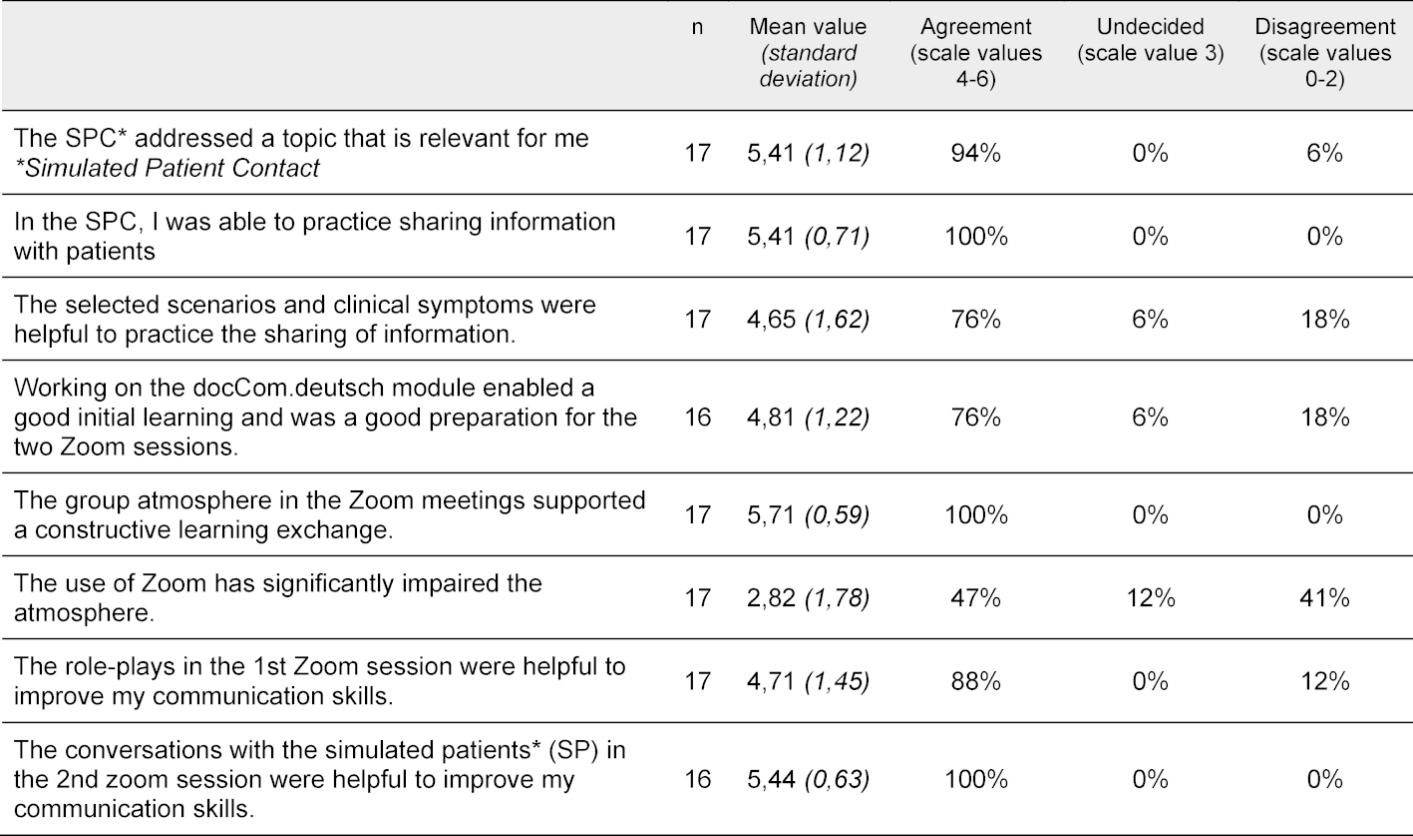
Evaluation results of the training course "Sharing information" as part of the 4^th^ semester communication curriculum
